# Vacuum-assisted closure (VAC®) systems and microbiological isolation of infected wounds

**DOI:** 10.1186/s13017-018-0216-z

**Published:** 2018-11-20

**Authors:** Valerio Cozza, Gilda Pepe, Marco Cintoni, Flavio De Maio, Giuseppe Tropeano, Sabina Magalini, Gabriele Sganga, Giovanni Delogu, Daniele Gui

**Affiliations:** 1 Emergency Surgery Department, Università Cattolica del Sacro Cuore, Fondazione Policlinico A. Gemelli IRCCS, Rome, Italy; 2 Microbiology Department, Università Cattolica del Sacro Cuore, Fondazione Policlinico A. Gemelli IRCCS, Rome, Italy; 3 Istituto di Clinica Chirurgica (9B) Catholic University of Sacred Heart, Largo Francesco Vito, 1, 00168 Rome, Italy

**Keywords:** Negative pressure wound therapy, Infection control, Wound infection, Wound contamination

## Abstract

**Background:**

Negative pressure wound therapy is now largely used to treat infected wounds. The prevention and reduction of healthcare-associated infections is a high priority for any Department of Health and great efforts are spent to improve infection control systems. It is assumed that vacuum-assisted closure (VAC®) dressings should be watertight and that all the secretions are gathered in a single container but there is no consistent data on air leakage and possible dispersion of bacteria from the machine.

**Methods:**

We have conducted a prospective experimental study on 10 patients with diagnosis of wound infection to verify whether the filtration process is microbiologically efficient. We compared the bacteria population present in the wound to the one present in the air discharged by the VAC® machine.

**Results:**

This study shows that the contamination of the VAC® machine is considerably lower than the environment or wound contamination.

**Conclusions:**

Negative pressure wound therapy system does not represent a risk factor for healthcare-associated infections.

## Introduction

Vacuum-assisted closure (VAC®) therapy is a system which promotes open wound healing through the application of negative pressure (negative pressure wound therapy, NPWT) [1–3], especially in infected tissues [4–7]. When applying negative pressure onto the bed of the wound, fluid material is removed, formation of granulation tissue is promoted, and wound edge approximation is helped [8]. These mechanisms are effective in promoting the healing process which would be otherwise difficult to treat, leading not only to economic advantages, but especially to a noticeably improved patients’ health [9].

It is assumed that VAC® dressings should be watertight and that all the secretions are gathered in a single container. Actually, a minimum leakage of air cannot be avoided completely. The air is vacuumed, filtrated, and discharged from the machine into the environment. Moreover, filters used in this process cannot be replaced by medical staff.

The object of this study is to verify whether the filtration process is microbiologically efficient. We compared the bacteria population present in the wound to the one present in the air discharged by the unit.

## Materials and methods

For this study, we enrolled 10 patients with abdominal wound or soft tissue infections (see Table 1), all treated with VAC® therapy. All the patients considered in the study were adult, hospitalised from February 2016 to April 2017 in the Emergency Surgery Unit at University Hospital “A. Gemelli”, Rome. The inclusion criteria were as follows: full thickness wound (involving subcutaneous fat down to fascia without extension to muscles or intra-abdominal organs), larger than 10 cm and producing at least 200 ml/day of exudate (in 7/10 cases), and with a verified positivity of bacterial culture (from the wound) [10].

**Table 1:** All the microbiological results

Study no.	Age	Male/female	LOS (days)	Site of VAC application	Inner machine plate		Injury swab		Environmental plate		Machine swab	
					P/N	Identified microorganisms	P/N	Identified microorganisms	P/N	Identified microorganisms	P/N	Identified microorganisms
1	27	M	74	Thigh abscesses	*Y*	*S. hominis*	*Y*	*C. striatum* *A. baumannii*	*Y*	*S. hominis* *S. haemolyticus* *A. baumannii*	*Y*	*E. faecium* *A. baumannii (1 colonia)*
2	27	M	74	Thigh abscesses	*Y*	*S. hominis*	*Y*	*E. coli (1 colonia)*	*Y*	*S. hominis*	*Y*	*S. hominis*
3	65	F	26	Sacral pressure ulcers	N		*Y*	*A. baumannii* *P. mirabilis* *P. stuartii*	*Y*	*S. epidermidis (1 colonia)*	N	
4	67	F	45	Necrotizing fasciitis	N		N		*Y*	*B. pumilus* *E. faecalis* *S. hominis*	N	
5	72	F	13	Sacral pressure ulcers	N		*Y*	*A. baumannii* *P. mirabilis* *E. faecium*	*Y*	*B. pumilus* *S. aureus* *P. mirabilis*	*Y*	*A. baumannii (1 colonia)*
6	48	M	62	Median laparotomy	N		*Y*	*A. baumannii*	*Y*	*S. hominis* *S. epidermidis* *B. simplex*	N	
7	70	M	21	Median laparotomy	N		*Y*	*A. baumannii*	*Y*	*A. baumannii* *S. hominis*	N	
8	66	M	37	Median laparotomy	N		*Y*	*A. baumannii*	*Y*	*S. haemolyticus* *S. hominis* *S. aureus*	N	
9	79	F	11	Median laparotomy	N		N		*Y*	*S. hominis* *E. faecalis*	N	
10	58	F	15	Enterocutaneous fistula	N		*Y*	*S. epidermidis*	*Y*	*S. hominis* *B. megaterium* *E. faecalis*	N	

*A. baumanii* Acinetobacter baumanii, *B. megaterium* Bacillus megaterium, *B. pumilus* Bacillus pumilus, *B. simplex* Bacillus simplex, *C. striatum* Corynebacterium striatum, *E. coli* Escherichia coli, *E. faecalis* Enterococcus faecalis, *E. faecium* Enterococcus faecium, *P. mirabilis* Proteus mirabilis, *P. stuartii* Providencia stuartii, *S. aureus* Staphylococcus aureus, *S. epidermidis* Staphylococcus epidermidis, *S. haemolyticus* Staphylococcus haemolyticus, *S. hominis* Staphylococcus hominis

### Study population description

Our study population was composed of 10 people, 5 males and 5 females. The average age was 57.9 years (median age 65.5 years, 27–79). VAC® was used in different types of wounds: 2 thigh abscesses, 2 sacral pressure ulcers, 4 infected median laparotomy, 1 necrotizing fasciitis, and 1 enterocutaneous fistula. As the majority of the patients admitted in our service, even among this study population, many were affected by multiple comorbidities (both acute and chronic). The most frequent were found to be cardiovascular and metabolic diseases, followed by malnourishment and bedridden syndrome. All of these promote bacterial superinfection, the infection persistence, delaying, consequently, the healing process. Two patients were otherwise healthy. Table 1 reports the demographics of the patients.

### Methodology of VAC® application and microbiological assays

The machine used was the VAC Ulta™ with standard GranuFoam™ dressing (different sizes) which was usually changed every 48/72 hours (h).

**Fig. 1:**
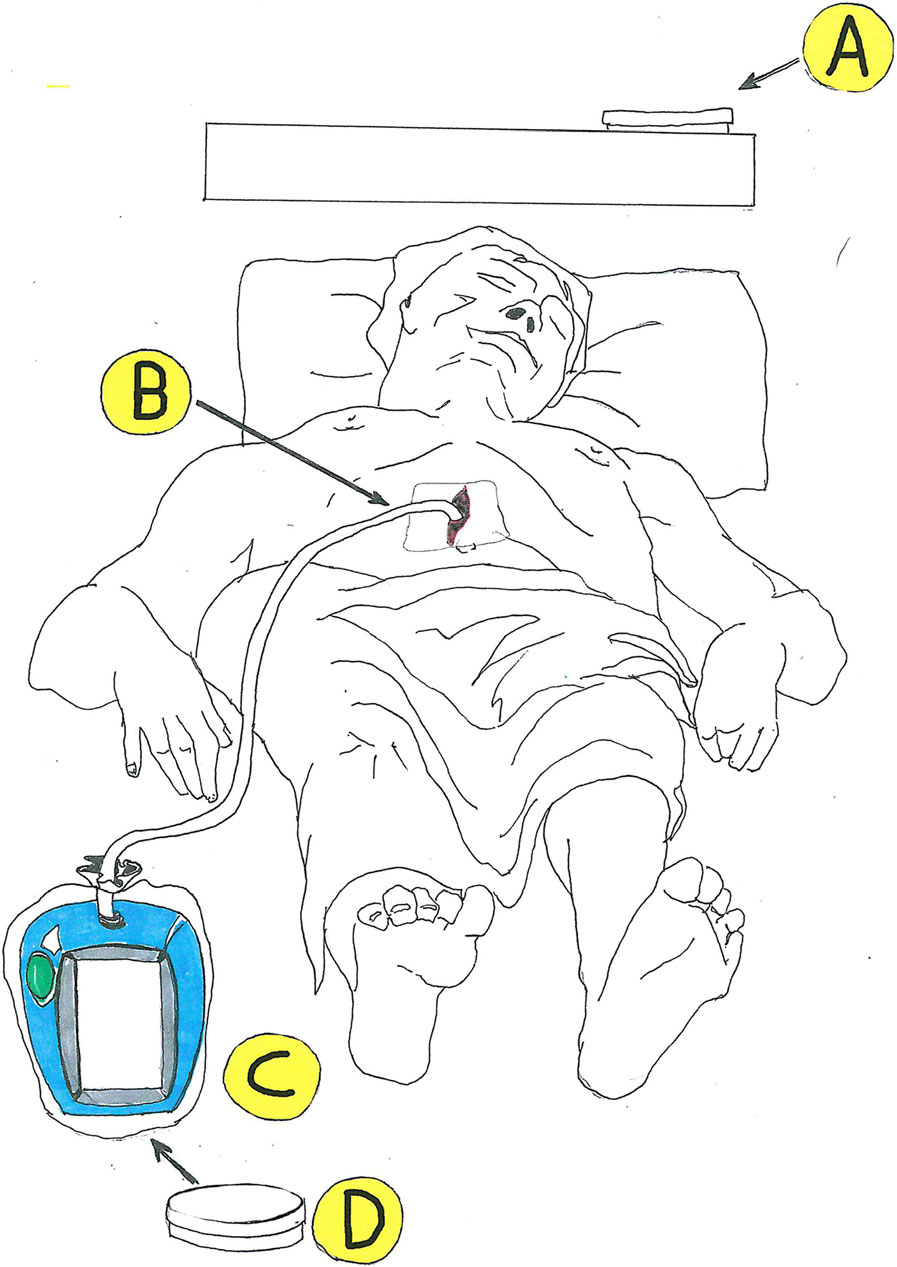
The four different specimens taken for the study. **a** Environment sample, a petri dish on the shelf. **b** Specimen collected from patient’s wound at the time of dressing change. **c** Specimen scrubbed from the VAC® unit *bodywork*. **d** Specimen from a Petri dish collocated inside the bag

In order to assess the eventual microbiological contamination of the air discharged from the VAC® unit, the following experimental study was designed: a sterile plastic bag was closed around the VAC® unit in a loose manner. As we can see from Fig. 1, the bag was sealed around the VAC® suction tube and the power cable. Data were collected through the microbiological examination of four different samples in a single 12-h span period (see Fig. 1): a specimen from a Petri dish in the patient room—usually at the head of the bed (to sample the environmental contamination) (a); a specimen collected from patient’s wound at the time of dressing change (b); a specimen scrubbed from the VAC® unit bodywork, i.e. the screen and the handle of the machine (c); and a specimen from a Petri dish collocated inside the bag (d).

The environmental plate was positioned on a shelf over the patient’s bed, high above the head. The machine swab was obtained rubbing a swab on the *canister* attached to the unit, as well as on the screen and handle of the device (considered to be the parts of the machine most frequently touched).

During the study, all the machines have worked correctly and no air leak was detected.

Specimens of the wound and the machine bodywork were plated on Trypcase Soy Agar (TSS, bioMérieux) and MacConkey agar (bioMérieux) and incubated overnight at 37 °C, supplemented with 5% CO_2_. Plates arising from the bag and from the environment were incubated in the same growth condition. Finally, single colonies of the positive specimens were identified using MALDI-TOF instrument (Bruker Daltonics) according to the manufacturer’s procedure.

To verify the object of the study, the bacterial growth from a swab of the wound was compared with the isolated colonies derived from the Petri dishes inserted in the bag next to the machine.

## Results

Table 1 shows the results. The injury swab (i.e. the swab taken from the wound) was positive in 80% of patients. In three patients, Gram-positive bacteria were isolated (*Corynebacterium striatum*, *Enterococcus faecium* and *Staphylococcus epidermidis*), whereas in seven patients Gram-negative bacteria were isolated. In particular, six out of seven patients were positive for *Acinetobacter baumannii;* of these, three presented a co-infection with *C. striatum*, *Proteus mirabilis* and *Providencia stuartii*, and *P. mirabilis* and *E. faecium* respectively.

The inner machine plate (i.e. taken inside the bag) resulted positive in 20% of patients, and Gram-positive bacteria were isolated in two patients (*Staphylococcus hominis*).

All environmental plates resulted positive. Gram-positive bacteria were present in the plates derived from all 10 patients and the most represented bacteria were *S. hominis* (70%), followed by *Enterococcus faecalis* (30%), *S. epidermidis*, *Staphylococcus aureus* and *Staphylococcus haemolyticus* (20%). Eighty percent of the patients had co-infection. Among these, the most frequent was *S. hominis* and *Streptococcus faecalis* (30%). Conversely, Gram-negative bacteria were isolated in 20% of our population (*A. baumannii*).

Machine swab (i.e. taken on the machine case) resulted positive in 30% of patients. Gram-positive bacteria were identified in two patients (*S. hominis* and *E. faecium*), as well as Gram-negative bacteria (*A. baumannii*).

## Discussion

Infections carry a heavy weight of intra-hospital deaths and costs, and there is a high level of attention towards aspects such as limitation of cross-contamination and reducing antibiotic treatments. A worldwide campaign has been conducted in many hospitals, including ours, to raise the attention on handwashing, which has shown to be one of the few very effective means to reduce nosocomial infections due to multi-resistant strains [11–13].

Extending the use of negative pressure wound therapy could potentially help in this sense: secreting wounds, in particular if infected, require multiple dressing changes, sometimes daily, whereas a NPWT dressing change can last for even more than 48 h. Furthermore, suction in the NPWT reduces pooling of fluid, which can itself facilitate bacterial growth. The dressing film is waterproof and therefore it does not allow pathogens from the skin to enter the wound. Additionally, some studies have shown that the NPWT could stop pathogens such as *S. aureus* from creating their biofilm [14].

The VAC® machine tolerates a minimal air leak before the alarm sets off; however, the amount of this leak is not known. The machine has a filter, which cannot be replaced by the medical staff; likely, it is replaced during the revision process that all the machines undergo periodically.

The rationale of this study is that the filter itself could represent a risk factor for environment or interhuman contamination. In fact, a large amount of infected fluid remains inside the canister and inside the system and potentially could be spread inside the environment. If so, we should radically change our approach to VAC® dressing changes to avoid potential contamination.

Our study shows that contamination in the air inside the bag containing the VAC® machine and the VAC® is considerably lower than the environment or wound contamination. This means that the cleaning work of the filter is successful, as shown by the fact that just 20% of the “inner machine plate” were positive.

The change of wound dressing represents a risk for environment contamination as well as the interhuman one [15–17]. Some of the pathogens isolated in the wounds are probably only contaminating bacteria; however, the percentages would suggest that this kind of dressing is not a risk factor for healthcare-associated infections.

This is a small study, and a larger group of patients is needed for a more significant statistical analysis. A larger study, better if multicentric, considering the heterogeneity of clinical practice (materials and methods used for wound treatment and dressing change) would indicate results reflecting different hospital realities. Anyway, this would require many resources and an excessively long time for the results’ publication which, in our case, seem to be very interesting from a microbiological point of view and promising from a clinical perspective as well. Furthermore, this data can be interpreted as a demonstration that the advanced wound dressing in our service is effective.

## Conclusions

The negative pressure wound therapy (NPWT), as analysed in our study, does not increase the risk of infection transmission: in fact, the contamination of the VAC® machine is considerably lower than the environment or wound contamination.

## Abbreviations

NPWT: Negative pressure wound therapy
VAC®: Vacuum-assisted closure
